# Plasma volume variation across the menstrual cycle among healthy women of reproductive age: A prospective cohort study

**DOI:** 10.14814/phy2.14418

**Published:** 2020-04-23

**Authors:** Sixtus Aguree, Hilary J. Bethancourt, Leigh A. Taylor, Asher Y. Rosinger, Alison D. Gernand

**Affiliations:** ^1^ Department of Nutritional Sciences The Pennsylvania State University University Park PA USA; ^2^ Department of Biobehavioral Health The Pennsylvania State University University Park PA USA; ^3^ Department of Anthropology The Pennsylvania State University University Park PA USA

**Keywords:** blood volume, indocyanine green, menstruating women, ovarian cycle, plasma volume

## Abstract

Increases in reproductive hormones like estrogen, play an important role in the remarkable increases in plasma volume observed in pregnancy. Accurate estimates of plasma volume expansion during pregnancy depend on correctly timing and measuring plasma volume in nonpregnant women. However, to date, there is no consensus on the pattern of plasma volume across the menstrual cycle. We prospectively measured plasma volume in 45 women across a single menstrual cycle. A urine‐based fertility monitor was used to time three clinic visits to distinct points in the menstrual cycle: the early follicular phase (~day 2), periovulation (~day 12), and the mid‐point of the luteal phase (~day 21)—based on a 28‐day cycle length. Healthy women aged 18–41 years with regular menstrual cycles and a healthy body weight were enrolled in the study. At each visit, blood samples were collected before and after injection of 0.25 mg/kg body weight of indocyanine green dye (ICG). Pre‐ and post‐ICG injection plasma samples were used to measure plasma volume. Preinjection samples were used to measure ovarian hormones and plasma osmolality. Mean plasma volume was highest during the early follicular phase (2,276 ± 478 ml); it declined to 2,232 ± 509 ml by the late follicular phase and to 2,228 ± 502 ml by the midluteal phase. This study found that overall variations in plasma volume are small across the menstrual cycle. Therefore, in clinical practice and research, the menstrual cycle phase may not be an important consideration when evaluating plasma volume among women of reproductive age.

## Graphical abstract

In this study, we timed plasma volume measurements based on the pattern of change in estrogen and progesterone concentrations, across the menstrual cycle. We showed that plasma volume remained stable across the menstrual cycle irrespective of the concentration of estrogen and progesterone. Our data established that plasma volume measurements can occur at any timepoint across the menstrual cycle, and that estimates are not impacted by physiological fluctuations observed in estrogen and progesterone concentrations in women of reproductive age.

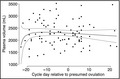

## 1 INTRODUCTION

Plasma volume expansion is an important physiological adaptation in a healthy pregnancy, with a 42%–50% increase between the nonpregnant state and the third trimester (Aguree & Gernand, 2019b; Chesley, 1972; Hytten, 1985; Lund & Donovan, 1967). Plasma volume changes during pregnancy are part of an adaptive physiologic response needed for hemodilution and a successful pregnancy outcome. Increasing levels of estrogen and progesterone, as well as changes in the renin‐angiotensin‐aldosterone system (RAAS), play a role in the increase in plasma volume across gestation. Compared with normal plasma volume, women with low plasma volume are at an increased risk of gestational hypertension, recurrent preeclampsia, recurrent pregnancy loss, and preterm delivery (Aardenburg, Spaanderman, Eijndhoven, Leeuw, & Peeters, 2006; Aardenburg et al., 2003; Donckers et al., 2012; Scholten et al., 2011, 2015; Spaanderman et al., 2001; Stekkinger, Scholten, Heidema, & Spaanderman, 2015). While plasma volume expansion in pregnancy has long been established, our knowledge of plasma volume across the menstrual cycle is limited. Previous reviews have reported mean nonpregnant plasma volumes of ~2,500–2,600 ml or 49 ml/kg (Aguree & Gernand, 2019b; Chesley, 1972; Croall, Sherrif, & Matthews, 1978; Lund & Donovan, 1967; Taylor & Lind, 1979) but menstrual phases were usually not specified. Knowledge of whether plasma volume fluctuates during the menstrual cycle could provide the basis for accurately estimating expansion across pregnancy as well as a better understanding of the menstrual cycle itself.

As in pregnancy, estrogen and progesterone‐related changes in the menstrual cycle may also regulate extracellular fluid and, thereby, lead to plasma volume expansion during specific phases of the cycle (Fortney et al., 1988; Stachenfeld & Taylor, 2005). The few studies that have examined plasma volume at different phases of the menstrual cycle have reported inconsistent results. For instance, in one study among a small group of healthy controls (*n* = 10) and formerly preeclamptic women (*n* = 39), plasma volume was higher during the midluteal phase compared to the early follicular phase – in both groups of parous women (Spaanderman et al., 2000). Of note, the mean plasma volume was lower in a subset of the formerly preeclamptic women (symptom‐free at the time of the study (*n* = 12)) compared to healthy controls. In contrast, another study aimed at investigating changes in plasma volume across the first trimester of pregnancy, but also conducting prepregnancy measurements, found that mean plasma volume was significantly higher during the early follicular phase relative to both the late follicular and midluteal phases (Bernstein, Ziegler, & Badger, 2001). In a series of plasma volume estimations across the menstrual cycle based on hemoglobin and hematocrit data, a nonsignificant decrease of 4.1% ± 8.9% was observed between the early follicular phase and late follicular phase (Cullinane, Yurgalevitch, Saritelli, Herbert, & Thompson, 1995).

These inconsistencies in previous studies may be due to small sample sizes, not timing plasma volume measurement based on reproductive hormone changes, and calculating plasma volume based on prediction equations instead of measurements based on dyes or tracers. Furthermore, these studies had different objectives and were conducted among women with different reproductive histories. We hypothesized that plasma volume would vary across the menstrual cycle due to increases in estrogen and progesterone concentrations, in the late follicular phase and the midluteal phase respectively. Our primary objective was to examine plasma volume prospectively across the menstrual cycle to evaluate within‐person change in measured plasma volume between three timepoints of the menstrual cycle. Our secondary objective was to assess the relationship between plasma volume and plasma osmolality, blood pressure, anthropometry, and reproductive hormones.

## 2 MATERIALS AND METHODS

### 2.1 Ethical approval

The study protocol was approved by the Office for Research Protections (ORP) at The Pennsylvania State University (STUDY00008383) and participating women signed informed consent. The trial is registered at http://www.clinicaltrials.gov (NCT03422809).

### 2.2 Study design and population

This study followed healthy eumenorrheic women across one complete menstrual cycle between January and November 2018. Participants were recruited from the State College area by a range of methods at The Pennsylvania State University, including platforms such as StudyFinder and ResearchMatch, and newspaper advertisements. Inclusion criteria for the study were as follows: (1) female, (2) age 18–44 years, (3) regular menstrual cycle (26–35 days), (4) general good health (does not have a known, ongoing health condition/medical issue that requires regular monitoring by a doctor or regular visits to the hospital), (5) BMI from 18.5 to 24.9 kg/m^2^, (6) nonsmoking, (7) nonpregnant, and (8) if ever pregnant, last pregnancy was ≥12 months ago. Participants were excluded if any of the following applied: (1) known allergy to shellfish or iodine, (2) blood pressure on the day of measurements was low or high (SBP <90 or ≥130 mmHg and/or DBP <60 or ≥80 mmHg), (3) current hypertension or previous hypertensive disorder in pregnancy (gestational hypertension or preeclampsia), (4) taking regular physician‐prescribed medication(s) for a health condition, (5) were trying to conceive, (6) using hormonal birth control (within the past 3 months, or used depot medroxyprogesterone acetate (DMPA) in the past 12 months), (7) diagnosed with polycystic ovary syndrome, or 8) breastfeeding. Interested persons were prescreened via telephone. Those potentially eligible were scheduled to visit the Clinical Research Center (CRC), a service unit of The Pennsylvania State University, for in‐person screening, informed consent, and enrollment into the study. A total of 47 women were enrolled in the study.

### 2.3 Tracking the menstrual cycle, study visits, and sample collection

Plasma volume was measured at three time points within a single menstrual cycle: the early follicular phase (EFP; ~day 2), the late follicular phase (LFP; ~day 12), and the midluteal phase (MLP; ~day 21). The visits were timed to correspond to phases of (1) low estrogen and progesterone concentrations, (2) high estrogen concentration, and (3) high progesterone concentration respectively.

During the first visit, participants completed a short questionnaire about their demographic characteristics and health and pregnancy histories. They were also provided with a fertility monitor (Clearblue Fertility Monitors Swiss Precision Diagnostics GmbH, Procter and Gamble, Cincinnati, OH), and PREGMATE Ovulation test strips. Women were instructed on how to use the fertility monitor to track daily changes in urinary hormone concentrations, starting at cycle day 6; the process involved collecting the first morning urine sample upon waking each morning, dipping a fresh test strip into the urine, and inserting it into the fertility monitor. The monitor displays results within 5 min and automatically stores the results. For each measurement, the monitor internally estimates the concentration of urinary estrone‐3‐glucuronide (E3G, the dominant estradiol in urine) and LH and displays one of three results, “low”, “high”, or “peak” fertility, which corresponds to low estrogen and low LH concentrations, high estrogen concentration, or high LH concentration respectively.

When the fertility monitor read “high”, the participants added daily testing with PREGMATE Ovulation test strips while continuing to test with the fertility monitor. The ovulation test strips were used as a backup to ensure that peak LH was detected (i.e., if it did occur but was not detected by the fertility monitor). Participants recorded daily test results on a form, and results were cross‐checked by research staff when the monitor was returned at the end of the study. Participants were scheduled for visit 2 as soon as possible after the monitor indicated the participant was at “high” fertility, usually within 2–3 days of “high” fertility, and before an indication of “peak” fertility. If a participant never reached “high” on the fertility monitor, she was dropped from the study, as this is indicative of an anovulatory cycle and resulted in a lack of the information on estrogen levels required to schedule the second visit.

Visit 3 was scheduled to occur 9 days after peak fertility readings on the monitor or test strips. We established this timeframe based on the knowledge that ovulation occurs 0.81 days following the LH surge (Johnson et al., 2015), and pregnanediol‐3‐ glucuronide (P3G, the major metabolite of progesterone) peaks 7.65 days after ovulation (Roos et al., 2015). If peak fertility was not detected, we scheduled the third visit 11–12 days after the indication of high fertility.

Prior to each clinic visit, participants were instructed to refrain from intense physical exercise for 24 hr and to remain well hydrated. Participants were instructed to arrive for each clinic visit after a 12‐hr overnight fast (water only); we specifically emphasized no alcohol or caffeine during the fast. After arrival, participants provided a fresh urine sample to test for pregnancy using QuickVue pregnancy test (an early pregnancy detection test for human chorionic gonadotropin in urine).

Body weight was measured to the nearest 0.1 kg on a digital scale, and height was measured to the nearest 0.1 cm with the use of a stadiometer (height at visit 1 only), both by trained study staff. We established a criterion, a priori, to drop women from the study if weight changed by >10% between visits (to exclude those with extreme weight change), however, the highest weight change was 3.6% (mean weight change was 0.8%) and no one was dropped for this reason. Body fat percentage was measured using a segmental bioelectric impedance analyzer (Tanita BC534 Glass InnerScan Body Composition Monitor, Tanita Corporation of America Inc., Arlington Heights, IL). Blood pressure was measured with an Omron Blood Pressure Monitor following recommended procedures (Whelton et al., 2017).

After these measurements, participants were then instructed to lie on a bed in a supine position with a small heating pad over the inside of the arm selected for IV insertion. Just before IV insertion, a temporary tourniquet was applied to aid in identifying an antecubital vein. Blood samples from the IV were collected into a 6 ml trace element‐free vacutainer blood collection tube coated with K_2_ ethylenediaminetetraacetic acid (K_2_ EDTA) for plasma volume and plasma osmolality assays. Blood samples were also collected into EDTA‐free vacutainer blood collection tubes (trace element‐free) for hormone analyses. Additional samples were collected into EDTA‐containing 2 ml vacutainer tubes for whole blood complete blood count (CBC) assays. This was followed by a bolus injection of ICG through the IV line, which was then flushed with saline solution. Starting at 2 min after ICG injection, blood samples were collected into 3 ml EDTA‐containing vacutainer blood collection tubes every 45 s, up to 5 min, for a total of five draws at exactly (min: sec) 2:00; 2:45; 3:30; 4:15; and 5:00.

EDTA‐treated tubes were gently inverted 10 times immediately after collection. Tubes were centrifuged (within 10 min of collection) at 1,500 × g for 15 min (Quest Diagnostics Horizon Centrifuge Model 642E) to separate plasma from blood cells. The plasma samples were aliquoted into cryovials and transported to the laboratory for PV determination. EDTA‐free tubes were gently inverted five times immediately after collection, allowed to clot (~30–45 min), and centrifuged at the same conditions as above and aliquoted into microcentrifuge tubes. Aliquots of preinjection serum and plasma samples were stored at −80ºC within 90 min of blood collection and kept frozen until analysis.

### 2.4 Plasma volume, osmolality, and reproductive hormones

A detailed description of the method for plasma volume measurement is published elsewhere (Aguree & Gernand, 2019a). In brief, standard concentrations of 2.5 mg/L to 15 mg/L were prepared by mixing appropriate volumes of ICG solution and participant's plasma (obtained before ICG injection). 100 µl of each blank (preinjection plasma), standard solution, and plasma samples were pipetted into a 96‐well plate and read on an Epoch plate reader (BioTek Instruments, Inc.) powered by Gen5^TM^ Software, set to a wavelength of 805 nm. Samples were read in triplicate and the mean was calculated. A standard curve was constructed from the standard concentration and absorbance and used to estimate ICG concentration in the plasma samples (obtained after ICG injection). Sample concentrations were natural log‐transformed and plotted against the time they were collected. The concentration of ICG dye in plasma was extrapolated to the virtual time of complete mixing, *t* = 0. Measures of plasma volume were obtained by dividing the dose of ICG injected by the extrapolated plasma ICG concentrations. The mean within‐person coefficient of variation (CV) for this assay in this study was 2.2%. Plasma volume measurements were conducted in the Penn State Department of Nutritional Sciences and completed within 2 hours of blood collection.

Osmolality of preinjection plasma was measured in duplicate by freezing point‐depression osmometry with the use of the Osmo1 Micro‐Osmometer (Advanced Instruments) by Dr. Asher Rosinger's Water, Health and Nutrition Laboratory in the Department of Biobehavioral Health. The within‐person CV was <1%. Estradiol and progesterone were measured at the Women's Health and Exercise Laboratory at Penn State directed by Dr. Mary Jane De Souza using ELISA kits produced by Siemens for the Immulite (SAP #10702832 and SAP #10381128, respectively; Siemens Medical Solutions Diagnostics). Serum samples were run on the Diagnostics Product Corporation Immulite 2000 Analyzer (Siemens Medical Solutions Diagnostics). Samples were measured consecutively, within a single run to limit analytical variability. Inter‐ and intra‐assay CVs were 16.0% and 15.0%, respectively, for estradiol, and 13.2% and 12.5%, respectively, for progesterone.

### 2.5 Statistical analysis

Categorical descriptive variables were presented as frequencies (%). We assessed normality by testing the distribution of continuous variables against a normal distribution using the Shapiro‐Wilk test and by visual inspection of kernel density plots. Estradiol and progesterone were log‐transformed to achieve normality. Continuous variables were presented as mean ± *SD*. We visually examined the pattern of plasma volume across the cycle by using fractional polynomial regression and creating prediction plots. Cycle days were standardized to the presumed day of ovulation by adding 2 days to the day of LH surge recorded by the fertility monitor because it is expected that ovulation occurs within 2 days of LH surge for most women (Behre et al., 2000; Fehring, Schneider, & Raviele, 2006). A linear mixed‐effects model was used to estimate changes in plasma volume between the three timepoints. Plasma volume and menstrual phase (time of visit as a categorical variable) were included as fixed variables, and participant identification number was entered as a random effect variable to account for repeated measurements within an individual. Similar models were run for plasma osmolality, blood pressure, weight, BMI, body fat (%), estradiol, and progesterone. Changes from the early follicular phase to the late follicular phase, the early follicular phase to the midluteal phase, and the late follicular phase to the midluteal phase were estimated by running models again with a different timepoint as the reference.

In a second model (adjusted model), we controlled for potential confounding by adding participants' age, BMI, and race (classified as White non‐Hispanic, Black/African American non‐Hispanic, Hispanic, Asian, and mixed/others), plasma osmolality, estradiol and progesterone to the previously described model as fixed variables. We also assessed the plasma volume pattern across the cycle by identifying participants that exhibited substantial fluctuations across the menstrual cycle. We considered a threshold of ∆ ± 8% a significant change based on other published work (Feldschuh & Katz, 2007). In this analysis, plasma volume change between each visit in the menstrual cycle was categorized into three groups: reduced plasma volume (>8% decrease), little to no change (≤|8|% change), and increased plasma volume (>8% increase).

Finally, we examined relationships between the variables measured at each visit (plasma volume and plasma osmolality, blood pressure, and anthropometric variables), combining data across visits. Spearman's correlation was run to examine the correlation between pairs of variables; fractional polynomial regression (and prediction plots) was used to visually examine bivariate relationships between plasma volume and plasma osmolality, blood pressure, anthropometric measures, and estradiol and progesterone concentrations.

In a sensitivity analysis, we restricted analysis to women who completed all three study visits. As plasma volume may be influenced by age and parity (Campbell & MacGillivray, 1972), we performed additional sensitivity tests restricted to nulliparous women, and also for women aged 20–35 years (to avoid those younger and older). Finally, we dropped women that were presumed to not to have ovulated (progesterone concentration >5 ng/ml at midluteal phase; Ecochard et al., 2013; Howards et al., 2009; Leiva, Bouchard, Boehringer, Abulla, & Ecochard, 2015). All statistical analyses were conducted with the use of Stata Version 14 software (Stata Corp).

## 3 RESULTS

### 3.1 Study participation and characteristics of women

**Figure 1:**
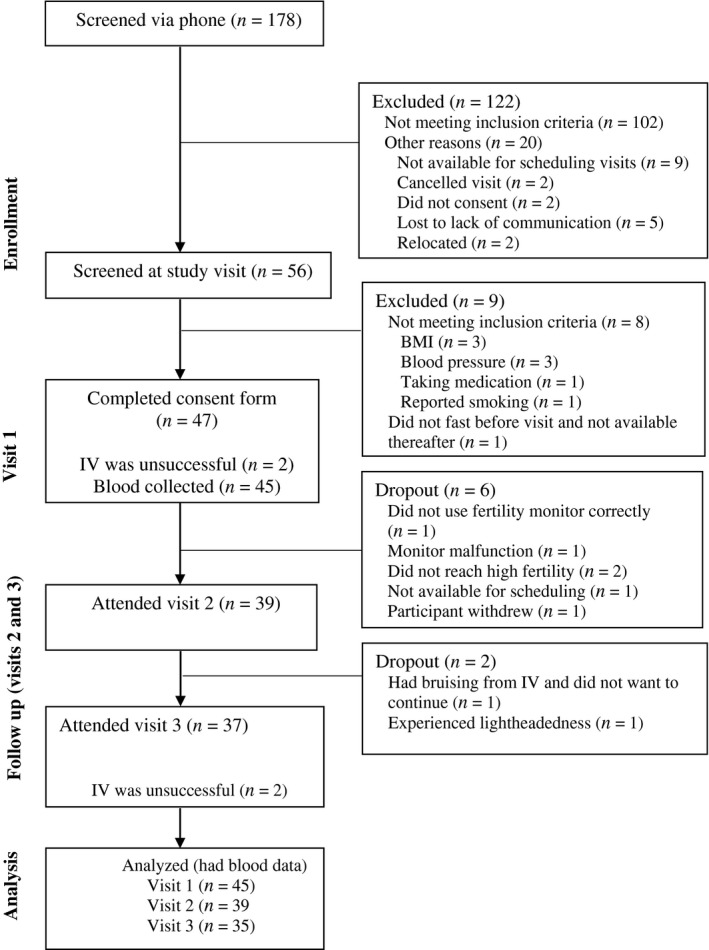
Flow chart of participants. IV; intravenous

A total of 178 women were screened, of which 47 women were enrolled into the study (Figure 1). At visit 1, blood samples were not collected for two women because the phlebotomist could not successfully establish an IV, and these subjects were dropped from the study. Therefore 45 women had a blood draw and ICG injection at visit 1, and this was our analytic sample. One plasma volume determination at visit 1 was unsuccessful due to an error in measurement; six participants were dropped between visits 1 and 2; two were dropped between visit 2 and visit 3; and two were dropped at visit 3 because the IV insertion was unsuccessful (Figure 1). Therefore, plasma volume is reported for 44, 39, and 35 women at visits 1, 2, and 3, with actual cycle day of visits 3.3 ± 0.9, 13.1 ± 2.7, and 23.8 ± 2.2 respectively. Participants were young, predominantly nulliparous, and mostly undergraduate students (Table 1). Cycle length recorded during the measured cycle ranged from 26 to 33 days. The mean difference in the menstrual cycle length between what was reported at enrollment (previous cycle) versus reported during the studied cycle was 0.94 ± 1.28 days.

**Table 1:** Baseline descriptive characteristics and menstrual cycle information of study participants (*n* = 45)^a^

Characteristics	Value^b^
Age, y	24.2 ± 5.6
Height, cm^c^	163.9 ± 7.0
Race, *n* (%)	
White non‐Hispanic	19 (42.2)
Black/African American non‐Hispanic	2 (4.4)
Hispanic	4 (8.9)
Asian	14 (31.1)
Multiple or others^d^	6 (13.3)
Socio‐economic status, *n* (%)	
Low	6 (13.3)
Middle	33 (73.3)
High	6 (13.3)
Educational status, *n* (%)	
At least bachelor's degree	18 (40.0)
Associate degree	3 (6.7)
Undergraduate students	24 (53.3)
Never married, *n* (%)	39 (86.7)
Nulliparous, *n* (%)	39 (86.7)
Menstrual cycle length, days	
Reported as last cycle before enrollment	28.9 ± 1.8
Recorded during study	28.0 ± 2.0

^a^ Self‐reported by the participant, unless otherwise indicated.

^b^ Mean ± *SD* or *n* (%) for categorical data.

^c^ Measured.

^d^ Two or more races (3); Others (2); Not specified (1).

### 3.2 Plasma volume changes and the associations with other participant characteristics

**Figure 2:**
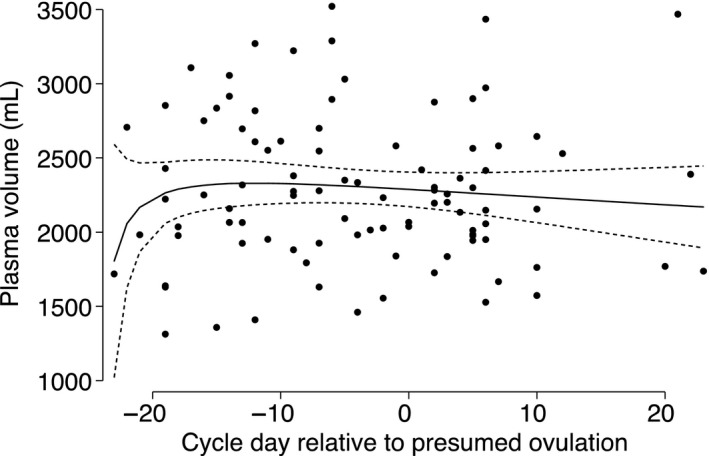
Fractional polynomial with regression (line) of plasma volume on cycle day relative to presumed date of ovulation. Number visits = 95. Dots represent data from study participants; solid line represents prediction based on all data; short dashed line represents the 95% CI around the prediction. Day of presumed ovulation was calculated as the date of LH surge recorded by the fertility monitor plus 2 days. Dates of measurements were subtracted from the presumed date of ovulation for each woman; cycle days before the presumed date of ovulation are negative (follicular phase) and cycle days after ovulation are positive (luteal phase)

Plasma volume estimates across all visits ranged from 1,211 to 3,520 ml with high variability at each visit (Table 2 and Table S1). There was a small decline, on average, in plasma volume across the menstrual cycle (Figure 2). Overall, the highest mean plasma volume (2,276 ± 476) was observed during the early follicular phase (Table 2). Plasma volume declined slightly to 2,232 ± 509 ml in the late follicular phase and then remained similar at 2,228 ± 502 ml in the midluteal phase. On average, plasma volume went down by 2.2% (*p* = .481) between the early and late follicular phase; 0.3% (*p* = .933) between the late follicular phase and the midluteal phase; and 2.4% (*p* = .536) between the early follicular phase and the midluteal phase. Among women with complete data, two showed a decrease in plasma volume >8%, and four showed an increase >8% from the early follicular phase to the midluteal phase.

**Table 2:** Means and within‐person differences in plasma volume and other key anthropometric and hormone measures across a single menstrual cycle

Variables	Mean estimates across the menstrual cycle			Mean change between visits^1^		
	EFP (*n* = 45)	LFP (*n* = 39)	MLP (*n* = 37)	LFP versus EFP	MLP versus LFP	MLP versus EFP
	Mean ± *SD*	Mean ± *SD*	Mean ± *SD*	β (95% CI)	β (95% CI)	β (95% CI)
Plasma volume^2^, ml	2,276 ± 476	2,232 ± 509	2,228 ± 502	−49 (−189, 88)	−6 (−149, 137)	−55 (−231, 120)
Plasma volume by weight, ml/kg	39 ± 6	38 ± 7	38 ± 9	−0.8 (−3.1, 1.6)	0.2 (−2.3, 2.6)	−0.6 (−3.5, 2.3)
Plasma volume by LBM, ml/kg	52 ± 9	51 ± 10	51 ± 12	−1.6 (−4.8, 1.6)	0.7 (−2.7, 4.0)	−1.0 (−4.9, 3.0)
Plasma volume by BSA, ml/m^2^	1,389 ± 237	1,362 ± 262	1,364 ± 295	−28 (−111, 55)	1.1 (−86, 88)	−27 (−130, 75)
Plasma osmolality^3^, mOsm/kg	299 ± 6^a^	297 ± 8^ab^	296 ± 7^bc^	−2.7 (−5.6, 0.2)	−0.7 (−3.8, 2.3)	−3.4 (−6.6, −0.3)*
Systolic blood pressure, mmHg	105 ± 7	104 ± 7	105 ± 7	−1.3 (−3.1, 0.5)	0.8 (−1.1, 2.8)	−0.5 (−2.8, 1.8)
Diastolic blood pressure, mmHg	69 ± 6^a^	67 ± 6^ab^	68 ± 5^bc^	−1.4 (−2.8, 0.01)	−0.2 (−1.7, 1.3)	−1.6 (−3.1, −0.10)
Weight, kg	58.3 ± 6.9	58.4 ± 6.6	58.2 ± 6.7	−0.08 (−0.30, 0.15)	−0.02 (−0.25, 0.22)	−0.09 (−0.32, 0.14)
BMI, kg/m^2^	21.7 ± 1.9	21.7 ± 1.9	21.5 ± 1.9	−0.03 (−0.11, 0.06)	−0.002 (−0.09, 0.08)	−0.03 (−0.11, 0.06)
Body‐fat percentage, %	25 ± 5	24 ± 6	25 ± 5	−0.78 (−2.03, 0.47)	0.83 (−0.48, 2.15)	−0.06 (−1.25, 1.36)
Estradiol (log)^4^, pg/ml	1.45 ± 0.15^a^	1.93 ± 0.31^b^	2.06 ± 0.21^c^	0.48 (0.39, 0.57)***	0.13 (0.04, 0.22)**	0.61 (0.51, 0.71)***
Progesterone (log)^4^, ng/ml	−0.25 ± 0.25^a^	−0.20 ± 0.31^ab^	0.85 ± 0.39^c^	0.05 (−0.09, 0.19)	1.05 (0.90, 1.19)***	1.09 (0.96, 1.23)***

Abbreviations: BSA, body surface area was estimated by using height and weight equation of Dubois (DuBois & DuBois, 1989); EFP, early follicular phase; LBM, lean body mass; LFP, late follicular phase; MLP, midluteal phase.

*p*‐value refers to maximum likelihood estimator.

^a,b,c^Mean values with different superscripts are significantly different from each other.

^1^ Model‐estimated mean difference (95% CIs) between the timed points in the menstrual cycle for the corresponding variables determined by linear mixed‐effects regression with individual‐level random intercepts. Corresponding variables (e.g., plasma volume) and menstrual phase were entered as fixed variables. Subject identification was included as a random effect variable to account for repeated measurements.

^2^ Plasma volume EFP (*n* = 44), LFP (*n* = 39), MLP (*n* = 35).

^3^ EFP (*n* = 43), LFP (*n* = 37), MLP (*n* = 34).

^4^ Measured in serum.

* *p* < .05.

** *p* < .01.

*** *p* < .001.

The highest mean plasma osmolality was also observed during the early follicular phase (Table 2). Overall, plasma osmolality decreased by 0.9% (*p* = .064) between the early and late follicular phase; 0.2% (*p* = .644) between the late follicular phase and the midluteal phase; and 1.1% (*p* = .034) between the early follicular phase and the midluteal phase. The ratio of plasma volume to plasma osmolality was consistent at 7.6 ± 1.8 ml mOsm kg^‐1^ across the menstrual cycle.

No change in systolic blood pressure was observed across the cycle, but diastolic blood pressure decreased by 1.6 mmHg (2.3%) between the early follicular phase and the midluteal phase (*p* < .036). By design, body weight and percent body fat were relatively constant across measurements. Estradiol concentration increased by 33% (*p* < .001) between the early and late follicular phase, 6% (*p* < .007) between the late follicular phase and the midluteal phase, and 42% between the early follicular phase and the midluteal phase (*p* < .001). Progesterone concentration increased by 20% (*p* = .492) between the early and late follicular phase, 526% (*p* < .001) between the late follicular phase and the midluteal phase, and 442% between the early follicular phase and the midluteal phase (*p* < .001).

**Figure 3:**
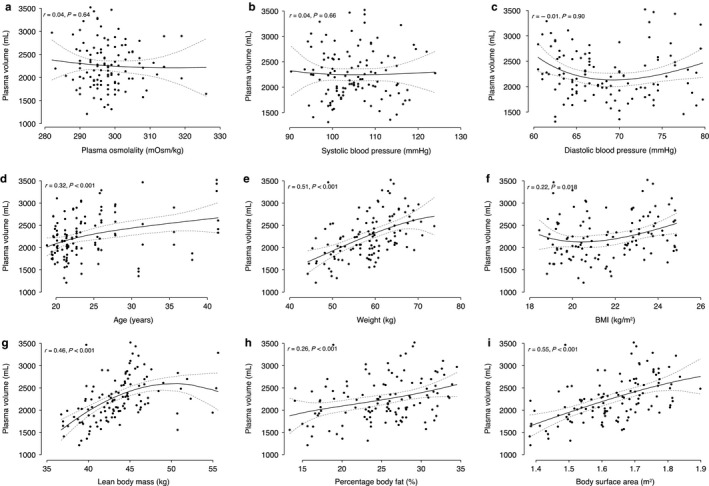
Fractional polynomial with regression (line) of plasma volume on plasma osmolality (a), systolic blood pressure (b), diastolic blood pressure (c), age (d), weight (e), BMI (f), lean body weight (g), body fat (%) (h), body surface area (i). Number visits = 118 (113 for plasma osmolality). Dots represent data from study participants; solid lines represent prediction based on all data; short dashed line represents the 95% CI around the prediction. Adjusting for lean body mass did not change results except for attenuating the relationship with BMI

**Figure 4:**
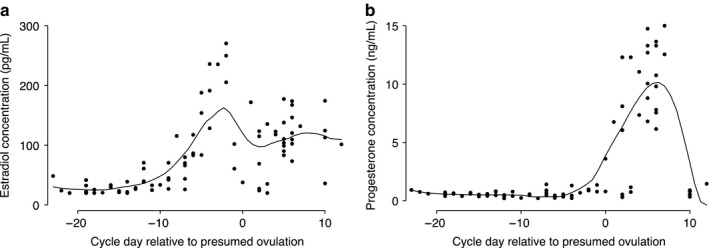
Locally weighted regression (line) of estrogen (a) and progesterone (b) concentrations versus cycle day from ovulation. Number visits = 95. Dots represent data from study participants; solid black line represents prediction based on all data. Day of presumed ovulation was calculated as the date of LH surge recorded by the fertility monitor plus 2 days. Dates of measurements were subtracted from the presumed date of ovulation for each woman; cycle days before the presumed date of ovulation are negative (follicular phase) and cycle days after ovulation are positive (luteal phase)

**Figure 5:**
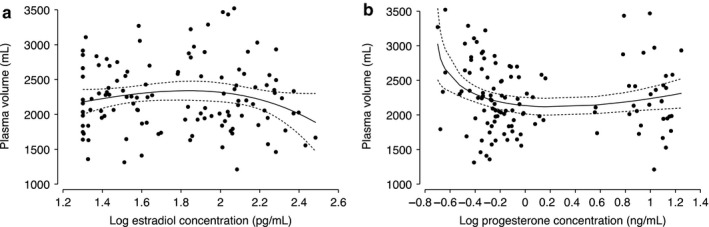
Locally weighted regression (line) of plasma volume versus log concentration of estrogen (a) and progesterone (b). Number visits = 118. Black dots represent data from study participants; solid gray line represents prediction based on all data; short dashed gray line represents the 95% CI around the prediction

Though plasma volume and plasma osmolality both went down across the menstrual cycle, the two were not associated (Figure 3). Plasma volume was also not related to blood pressure. However, plasma volume was strongly associated with anthropometric measures, particularly with BMI, body surface area, and lean body mass. The correlation between plasma volume and anthropometry measures ranged from a low of 0.22 (BMI) to a high of 0.55 (body surface area). Plasma volume was also positively correlated with age (*r* = .32). Change in plasma volume was weakly correlated with changes in plasma osmolality (*r* = −.13–.06, all *p > *.05), blood pressure (*r* = −.09–.25, all *p > *.05), and weight (*r* = −.08–.25, all *p* > .05; Table S2). Similarly low correlations were observed for plasma volume change and changes in BMI, body fat percentage and ovarian hormones. Estradiol and progesterone concentration from this study followed the normal expected pattern in a cycle (Figure 4). Neither estradiol nor progesterone concentrations were associated with plasma volume (Figure 5).

When we adjusted for women's age, BMI, race/ethnicity (White non‐Hispanic, Black/African American non‐Hispanic, Hispanic, Asian, and mixed/others), plasma osmolality, estradiol and progesterone, and time, plasma volume decreased by 1.0% (−23 [95% CI: −233, 186] ml, *p* = .828) between the early and late follicular phase and rose by 2.7% (61 [95% CI: −277, 399] ml, *p* = .725) between the early follicular phase and the midluteal phase (Table 2). We report the percentiles of the plasma volume distribution across all visits in Table S3.

### 3.3 Sensitivity analysis

Mean plasma volume values for women with complete data at all three study visits (*n* = 34) were 2,301 ± 510 ml (early follicular phase); 2,239 ± 538 ml (late follicular phase); and 2,233 ± 509 ml (midluteal phase), similar to the main findings (Table S1). Among the women who completed all study visits, plasma volume decreased by 2.7% (*p* = .428) between the early and late follicular phase; and 3.0% (*p* = .490) between the early follicular phase and the midluteal phase. The mean plasma volume for participants who dropped out of the study, or otherwise did not complete all three visits, was (*n* = 9) 2,244 ± 317 ml (early follicular phase; Table S1). Mean plasma volume estimates were comparable between participants who eventually dropped from the study and those who completed all three visits (*t* = 0.32, *p* = .752). As noted at the beginning of the results, even women who dropped out after the first visit were included in the main results.

Among women aged 20–35 years, the mean plasma volume was 2,295 ± 464 ml (*n* = 34), 2,217 ± 504 ml (*n* = 30), and 2,182 ± 486 ml (*n* = 27) at the early follicular, the late follicular, and the midluteal phases respectively. Plasma volume declined by 3.7% (*p* = .318) from the early to late follicular phase; 1.3% (*p* = .750) between the late follicular phase and the midluteal phase; and 4.9% (*p* = .285) from the early follicular phase to the midluteal phase. Mean plasma volume estimates were comparable between participants aged 20–35 years and the overall results (*t* = 0.18, *p* = .860). Among nulliparous women only, the mean plasma volume was 2,262 ± 473 ml (*n* = 38), 2,213 ± 477 ml (*n* = 34), and 2,157 ± 416 ml (*n* = 31) in the early follicular, the late follicular, and the midluteal phases respectively. Plasma volume declined by 3.3% (*p* = .309) from the early to late follicular phase; 2.0% (*p* = .560) between the late follicular phase and the midluteal phase; and 5.3% (*p* = .194) from the early follicular phase to the midluteal phase. The mean plasma volume among parous women was 2,381 ± 590 ml (*n* = 5), slightly higher but not statistically significantly different from the nulliparous women (*t* = 0.52, *p* = .603). Restricting analysis to women who were presumed to have ovulated did not affect the results.

## 4 DISCUSSION

In this prospective cohort of healthy women of reproductive age, we found that plasma volume showed a very small decrease across the menstrual cycle from the early follicular phase to the midluteal phase. A fifth of the women showed a substantial change (>8% up or down) in plasma volume between the early follicular phase and the midluteal phase. Plasma volume showed a strong positive correlation with body weight, lean body mass, and body surface area, but no correlations with plasma osmolality, blood pressure (systolic and diastolic blood pressure), or reproductive hormones (estradiol or progesterone). Though plasma volume measures often focus on changes during pregnancy, recent studies have shown that prepregnant plasma volume could be an important factor in predicting risk of recurrent preeclampsia, recurrent pregnancy loss, and risk of preterm delivery (Donckers et al., 2012; Scholten et al., 2011; Aardenburg et al., 2003, 2006; Donckers et al., 2012; Scholten et al., 2011, 2015; Spaanderman et al., 2001; Stekkinger et al., 2015).

The mean plasma volume estimates and variations in the current study are comparable to those of Bernstein et al. (Bernstein et al., 2001) – both studies found that plasma volume estimates at the early follicular phase were the highest. However, the Bernstein et al*.* study found a small rise between the late follicular phase and the midluteal phase, which was not observed in the present study. Plasma volume variability in the present study, as shown by the standard deviation, was also larger than the Bernstein et al*.* study which could be due to differences in the population. The participants in this study were relatively younger (mostly students) than those from Bernstein et al. (aged 29.7 ± 2.3 years). Spaanderman et al. reported a large increase of 13% in plasma volume between the follicular phase and the luteal phase (Spaanderman et al., 2000). Participants of the Spaanderman study were white women, largely preeclamptic with few healthy controls, slightly older (31 ± 2 years), and parous. The sample size for the healthy controls in that study was relatively small (*n* = 10) compared to the current study. Spaanderman et al. used radioiodine‐labeled human serum albumin (^125^I HSA) dilution method to measure plasma volume while Bernstein et al. measured plasma volume using Evans blue dye dilution method, compared to the ICG dilution method in the present study. In another study in which changes in plasma volume were calculated from hemoglobin and hematocrit values, a 4% reduction between the early and late follicular phase was observed (Cullinane et al., 1995). Similar to the current study, Cullinane et al. reported a wide within‐person variability, indicated by a standard deviation of 9%.

During pregnancy, there is an increase in systemic vasodilation and vascular capacity as a result of the increase in activity of the renin‐angiotensin‐aldosterone system (RAAS). This helps maintain blood pressure, retain water and salt, and reduce atrial natriuretic peptide concentrations. The loss of water and salt creates an underfilled vascular system (Sanghavi & Rutherford, 2014). In response, plasma volume increases by filling the vascular space created (Magness, Parker, & Rosenfeld, 1993). In nonpregnant women of reproductive age, previous studies have reported higher plasma renin, plasma renin activity, and aldosterone concentrations in the luteal phase compared to the follicular phase (Chidambaram et al., 2002; Davis, Gibson, Casley, & Brown, 2017). If the changes in plasma volume across the menstrual cycle were driven by RAAS, we would expect to find higher plasma volume in the luteal phase, as observed by Spaanderman et al. (Spaanderman et al., 2000). However, in our study and others (Bernstein et al., 2001; Cullinane et al., 1995; Pahwa, Seth, & Seth, 1998), plasma volume was higher in the early follicular phase.

Results from animal studies have shown that prolonged administration of estradiol‐17β in ewes leads to classical cardiovascular alteration associated with pregnancy, and increased plasma volume by 14% (Chidambaram et al., 2002; Davis et al., 2017; Magness et al., 1993). Other studies have also reported increases in plasma volume following the administration of estrogen and progesterone (Pecins‐Thompson & Wood, 1997; Stachenfeld & Taylor, 2005). In an experiment to examine the influence of exogenous and endogenous estrogen, Fortney et al. observed that, while administration of exogenous estrogen was shown to significantly attenuate plasma volume loss, menstrual cycle fluctuation of endogenous estrogen had only a small transient effect in healthy women (Fortney et al., 1988). Furthermore, estrogen regulates the expression of angiotensinogen genes (Gordon, Chin, & Shupnik, 1992) and influences plasma renin activity (Magness et al., 1993; Miller, Anacta, & Cattran, 1999; Sealey et al., 1994). We were surprised that plasma volume was somewhat lower during the times of expected estrogen and progesterone peaks in the current study, and that there was no correlation between plasma volume and these hormones. This suggests that the physiological influence of exogenous and endogenous estrogen and progesterone on plasma volume may be different in women of reproductive age.

Previous studies have reported relationships between plasma volume (or blood volume) and body dimensions or anthropometry, finding strong correlations with body surface area (Feldschuh & Katz, 2007; Hurley, 1975; Pearson et al., 1995), body weight (Feldschuh & Enson, 1977), and lean body mass (Boer, 1984), but weak‐to‐moderate positive correlations with height (Feldschuh & Enson, 1977) and BMI (Miller & Borlaug, 2020). The relationship between blood volume and weight is curvilinear in nature, with a higher volume/weight ratio in very thin individuals and lower ratio in obese individuals (Feldschuh & Enson, 1977) because fat does not increase blood volume as lean mass does. These relationships are consistent with the correlations between plasma volume and anthropometry measures reported in the present study.

Other characteristics that can influence plasma volume are race, parity, and age. Higher plasma volume has been reported in women of European origin compared to those of Indian and Andean ancestry (Hutchins, 1980; Vargas et al., 2007). In the present study, though plasma volume was slightly higher in white women compared to Asian women, these differences were not significant. Previous studies were typically conducted in parous women, whereas most of our participants were nulliparous. The independent influence of age on plasma volume is not often clearly delineated since parous women are also likely to be older than nulliparous women. In the current study, age was positively associated with plasma volume, even when limited to only nulliparous women and controlling for lean body mass. Overall, women in this study were mostly nulliparous and young. Previous studies have reported higher plasma volume in parous women compared to nulliparous, and multigravidae compared to primigravidae (Campbell & MacGillivray, 1972, 1977).

To examine hydration as a factor in plasma volume, we also measured osmolality. Our findings of reduced plasma osmolality across the menstrual cycle is consistent with that of Spruce et al., who found a reduction in plasma osmolality between the early follicular phase and the luteal phase (Spruce, Baylis, Burd, & Watson, 1985). Moderate increases/decreases in blood volume have been shown to be associated with a small change in plasma osmolality, with the process being tightly regulated by the antidiuretic hormone arginine vasopressin (Robertson & Athar, 1976). Neither plasma volume nor changes in volume were associated with plasma osmolality. Previous studies have reported that changes in plasma volume were associated with changes in plasma osmolality during intensive exercise (Jimenez et al., 1999), but the present study did not find any association between plasma volume and osmolality in a nonexercise setting, though plasma volume and osmolality both tended to decrease across the menstrual cycle.

Finally, we had only six women that had a >|8|% change in plasma volume. Although these numbers are under powered to detect differences (and therefore not part of the results), we observed that body weight and composition indicators were similar between women with large versus small changes, yet systolic and diastolic blood pressure were higher in those that had a change >8% and lower in those that had a change >−8%, compared to women with a change <|8|%. Examining characteristics of women with large swings in plasma volume across a menstrual cycle deserves attention in larger studies.

### 4.1 Strengths and limitations

The strengths of this study include a careful design to include healthy women with regular menstrual cycles and normal BMI. All visits were conducted at a similar time in the morning. We also used a fertility monitor to help us prospectively track and schedule participants’ visits, timed to specific phases of the menstrual cycle in order to reflect concentrations of key menstrual cycle hormones. In addition to the fertility monitor, participants were provided with LH test strips to help confirm ovulation. We instructed participants to be well‐hydrated before study visits to eliminate any effect of hydration on plasma volume, coupled with the measurement of three quantitative markers of hydration status.

This study also had limitations. Plasma volume measurement using ICG is somewhat invasive, and that might have prevented some women from participating in the study. Cycle length also varies with age, and considering the wide range of participants, 18–41 years, this included women in the early and late stages of the reproductive cycle. However, when we restricted data analysis to participants aged 20–35 years, the estimates for plasma volume were comparable to the whole sample. Most of the study participants were nulliparous, which may limit the applicability of these findings to parous women. It remains unclear if these findings would apply to overweight/obese and/or women with irregular menstrual cycles.

## 5 CONCLUSION

This is one of the first carefully controlled studies to measure how plasma volume changes across the menstrual cycle among healthy women using a novel dye methodology. Contrary with our hypothesis and previous work, our results suggest that plasma volume is relatively stable overall across the menstrual cycle in healthy eumenorrheic women, slightly lower in the late follicular phase and the midluteal phase compared to the early follicular phase. While we wait for these results to be confirmed, our data suggest that the menstrual cycle phase is not an important factor when evaluating plasma volume for clinical practice and research in women of reproductive age. Larger studies with several repeated follow‐ups in the same women within and across multiple cycles (including a wider range of body weight, parity, and race/ethnicity) would allow for better control of intra‐ and interindividual variation, are needed. Future work in nonpregnant women should also explore whether changes in plasma volume may vary for women with irregular menstrual cycles.

## CONFLICT OF INTEREST

The authors report no financial or other conflicts of interest relevant to this study.

## AUTHORS’ CONTRIBUTION

All authors took part in critical reading and revision of the manuscript. S.A: study conception, execution, laboratory analysis, and data analysis, interpretation and manuscript drafting. H.B.: Laboratory analysis, and interpretation. L.A.T.: study execution and laboratory analysis. A.Y.R.: study conception and interpretation. A.D.G.: study conception, execution, and interpretation.

## Supporting information



Table S1‐S3
