# Supplementary material for: Plasma volume variation across the menstrual cycle among healthy women of reproductive age: A prospective cohort study
Source: Physiol Rep. 2020 Apr 23;8(8):e14418. doi: 10.14814/phy2.14418 (PMC7178826; doi:10.14814/phy2.14418)
Supplement: Supplementary file 1 — Table S1‐S3 [file PHY2-8-e14418-s001.docx]

**Supplementary Table S1**

Mean plasma volume, and other key anthropometric and hormone measures comparing participants with complete data to those missing data at one to two time points.

|  | EFP | | | | | | p-value^2^ | p-value^3^ |
| --- | --- | --- | --- | --- | --- | --- | --- | --- |
|  | All enrolled women (n=45) | | Women completing 3 visits  (n=34) | | Women completing <3 visits (n=10) | |  |  |
| Variable | Mean ± SD | Range^1^ | Mean ± SD | Range^1^ | Mean ± SD | Range^1^ |  |  |
| Plasma volume ^4^, mL | 2276 ± 476 | 1313-3270 | 2301 ± 510 | 1313-3270 | 2244 ± 317 | 1646-2696 | 0.824 | 0.752 |
| Plasma volume by weight, mL/kg | 39 ± 6 | 26-52 | 39 ± 7 | 26-52 | 39 ± 5 | 34-51 | 1.000 | 1.000 |
| Plasma volume by LBM, mL/kg | 52 ± 9 | 34-72 | 53 ± 10 | 34-72 | 53 ± 6 | 45-64 | 0.644 | 1.000 |
| Plasma volume by BSA, mL/m^2^ | 1389 ± 237 | 873-1935 | 1399 ± 255 | 873-1935 | 1386 ± 152 | 1166-1685 | 0.859 | 0.885 |
| Plasma osmolality ^5^, mOsm/kg | 299 ± 6 | 286-319 | 300 ± 7 | 288-319 | 297 ± 5 | 286-301 | 0.505 | 0.236 |
| Systolic blood pressure, mmHg | 105 ± 7 | 94-124 | 106 ± 8 | 95-124 | 103 ± 6 | 96-112 | 0.556 | 0.280 |
| Diastolic blood pressure, mmHg | 69 ± 6 | 60-80 | 70 ± 6 | 60-80 | 66 ± 5 | 60-77 | 0.465 | 0.062 |
| Weight, kg | 58.3 ± 6.9 | 44.5-73.8 | 58.4 ± 6.8 | 44.5-71.0 | 58.0 ± 8.3 | 46.2-73.8 | 0.949 | 0.877 |
| BMI, kg/m^2^ | 21.7 ± 1.9 | 18.9-24.8 | 21.5 ± 1.9 | 18.9-24.8 | 22.1 ± 1.9 | 19.4-24.4 | 0.645 | 0.385 |
| Body-fat percentage, % | 25 ± 5 | 14-34 | 25 ± 5 | 14-34 | 26 ± 4 | 20-31 | 1.000 | 0.566 |
| Estradiol (log) ^6^, pg/mL | 1.45 ± 0.16 | 1.30-1.88 | 1.46 ± 0.17 | 1.30-1.88 | 1.41 ± 0.10 | 1.30-1.58 | 0.790 | 0.383 |
| Progesterone (log) ^6^, ng/mL | -0.25 ± 0.25 | -0.70-1.10 | -0.25 ± 0.28 | -0.70-1.10 | -0.24 ± 0.13 | -0.51-(-0.07) | 1.000 | 0.910 |

EFP, early follicular phase ; LBM, lean body mass ; BSA, body surface area was estimated by using height and weight equation of Dubois (DuBois & DuBois, 1989).

^1^ Minimum-maximum values

^2^ Comparison of estimates for women with total data vs complete data across study via independent t-test

^3^ Comparison of estimates for women with complete data vs incomplete data via independent t-test

^4^ Plasma volume (n=34 complete data; n=9 incomplete data).

^5^ Plasma osmolality (n=33 complete data; n=9 incomplete data).

^6^ Estradiol and progesterone were measured in serum.

**Supplementary Table S2**

Associations between within-person changes in plasma volume and changes in plasma osmolality, blood pressure, and body composition and ovarian hormones across the menstrual cycle (n = 34)^1^.

|  | LFP vs EFP | MLP vs LFP | MLP vs EFP |
| --- | --- | --- | --- |
|  | r (p) | r (p) | r (p) |
| ∆ Plasma osmolality | -0.13 (0.436) | 0.06 (0.740) | 0.06 (0.731) |
| ∆ Systolic blood pressure | -0.09 (0.610) | 0.15 (0.379) | 0.02 (0.900) |
| ∆ Diastolic blood pressure | 0.09 (0.611) | 0.25 (0.151) | 0.03 (0.880) |
| ∆ Weight | -0.08 (0.651) | 0.25 (0.142) | 0.08 (0.649) |
| ∆ BMI | -0.07 (0.657) | 0.22 (0.198) | 0.06 (0.745) |
| ∆ Body-fat percentage | -0.04 (0.826) | 0.01 (0.667) | 0.05 (0.764) |
| ∆ Estradiol (log) | -0.06 (0.711) | -0.15 (0.388) | -0.01 (0.969) |
| ∆ Progesterone (log) | -0.01 (0.956) | -0.03 (0.878) | 0.03 (0.872) |

PV, plasma volume; EFP, early follicular phase; LFP, late follicular phase prospective; MLP, Midluteal phase; LBM, lean body mass; BSA, body surface area.

^1^ Changes (%) between any two study visits (timepoints), women with plasma volume data across all three visits (n=34).

**Supplementary Table S3**

Descriptive summary of plasma volume (n = 45)^1^.

|  | Estimate (95% CI) (mL) |
| --- | --- |
| Plasma volume, mL |  |
| 5^th^ | 1528 (1321, 1642) |
| 10^th^ | 1646 (1535, 1747) |
| 20^th^ | 1837 (1723, 1949) |
| 25^th^ | 1925 (1770, 1998) |
| 50^th^ | 2206 (2064, 2300) |
| 75^th^ | 2564 (2403, 2706) |
| 80^th^ | 2663 (2542, 2856) |
| 95^th^ | 3222 (2924, 3462) |
| 99^th^ | 3468 (3284, 3521) |

^1^ Number visits = 118.
